# Supplementary material for: Ih Block Reveals Separation of Timescales in Pyloric Rhythm Response to Temperature Changes in Cancer borealis
Source: bioRxiv. 2024 Aug 6:2024.05.04.592541. Originally published 2024 May 7. Preprint. [Version 3] doi: 10.1101/2024.05.04.592541 (PMC11100622; doi:10.1101/2024.05.04.592541)
Supplement: 1 [file NIHPP2024.05.04.592541V3-supplement-1.pdf]

bioRxiv preprint doi: https://doi.org/10.1101/2024.05.04.592541; this version posted August 6, 2024. The copyright holder for this preprint (which was not certified by peer review) is the author/funder, who has granted bioRxiv a license to display the preprint in perpetuity. It is made available under aCC-BY 4.0 International license.

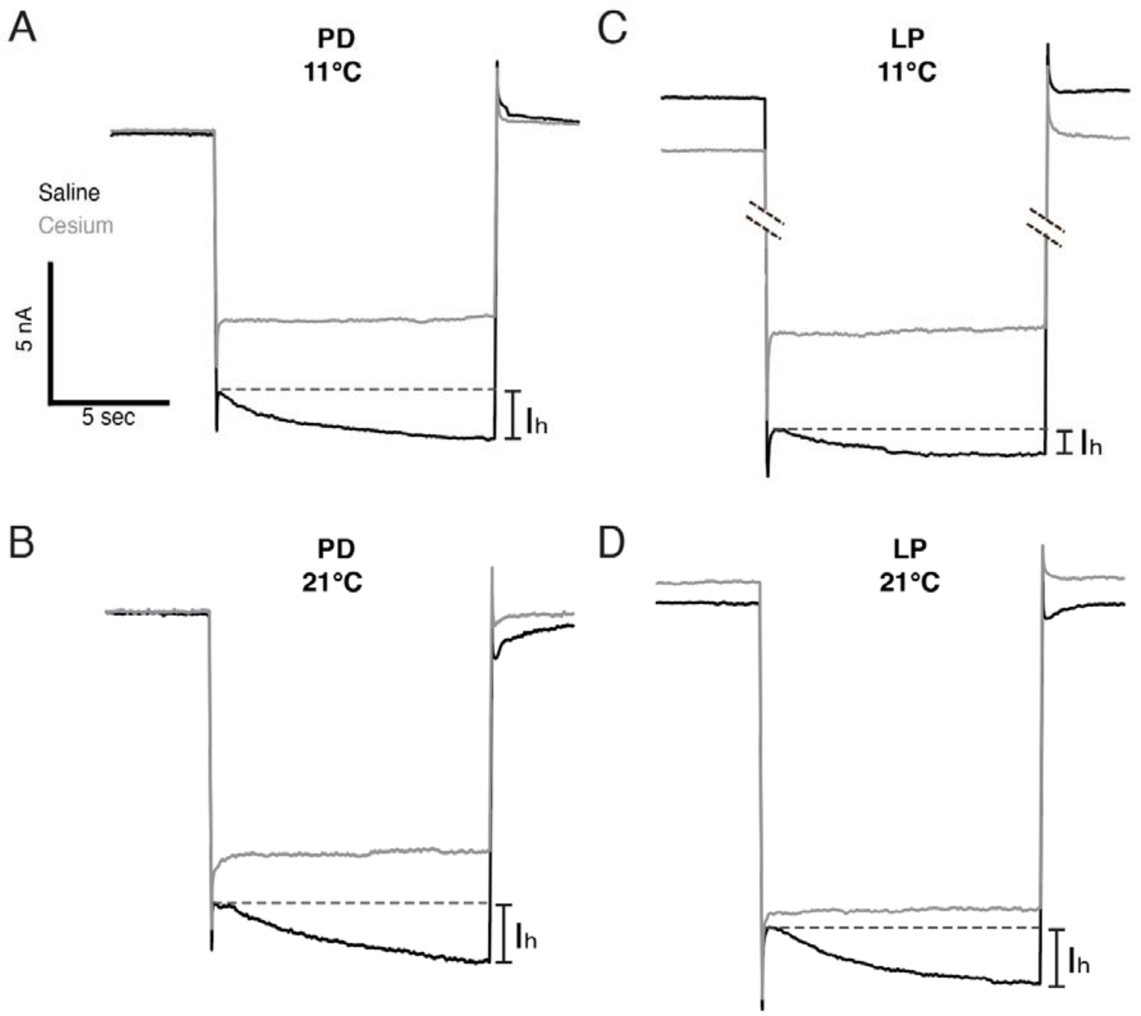

Figure 1—figure supplement 1: Voltage Clamp traces of PD and LP cells at the temperature extrema of these experiments in both saline and Cs^+. Cells were held at -50mV for 9 seconds, then -110mV for 12 seconds before being returned to -50mV for 4 seconds. In saline, there is a notable sag current (I_h) when the cell is held at -110mV (black traces, difference in initial and steady state current highlighted by dashed line). This sag current is absent when the cell is in Cs^+ (grey traces). **(A)** PD cell at 11°C. **(B)** PD cell at 21°C. **(C)** LP cell at 11°C. **(D)** LP cell at 21°C. Each cell is from a separate preparation.
